# Anterior disc derangement with reduction of the temporomandibular joint: a case report

**DOI:** 10.1186/s13256-018-1637-8

**Published:** 2018-05-27

**Authors:** Katie L. Crockett, Richard Bourassa, Tyler Friesen

**Affiliations:** 1 Bourassa and Associates Rehabilitation Centre, 109–294 Venture Cres, Saskatoon, SK S7K 6M1 Canada; 2 School of Physical Therapy, University of Saskatchewan, Saskatoon, SK Canada

**Keywords:** Temporomandibular joint dysfunction, Physical therapy, Platelet-rich plasma, Injection, Manual therapies

## Abstract

**Background:**

Temporomandibular dysfunction involving anterior disc derangement with or without reduction, secondary to posterior ligament insufficiency is typically managed conservatively with success in a majority of patients. When conservative management fails, the next step in the continuum of care is unclear. Platelet-rich plasma injection combined with a 3-week immobilization period may be effective in treating posterior ligament insufficiency following a period of physical therapy. The result of this case was exceptionally successful, with the patient reporting 100% improvement 6 months post-injection. Prior to this case, we predicted a 20% success rate based on her inability to maintain the effects of conservative management over the long term.

**Case presentation:**

A 33-year-old white woman presented with temporomandibular dysfunction, which responded to an initial course of physical therapy aimed at restoring the mechanics of her temporomandibular joint, exercise management, and education on self-management strategies. She returned 20 months later and responded well to another course of physical therapy. Despite improvement in pain, range of motion, and mechanics, she continued to present with a reduction click at the end range of opening. The crisp and loud nature of the reduction click indicated a viable posterior ligament and reduction of the anteriorly displaced disc. She opted for platelet-rich plasma injection, provided by a chronic pain specialist with the assistance of a physical therapist. She was immobilized for 3 weeks, followed by a weaning period with reduced posterior support for an additional 5 weeks. Follow-up appointments with a physical therapist occurred at 3 weeks, 8 weeks, and 6 months post-injection.

**Conclusions:**

At 6 months, she reported 100% improvement. Objectively, there was no indication that the disc condyle relationship was disrupted. At 6 months post-platelet-rich plasma injection, preceded by a period of conservative physical therapy management, and followed with appropriate physical therapy follow-up, this individual had complete resolution of her anterior disc derangement with reduction.

**Electronic supplementary material:**

The online version of this article (https://doi.org/10.1186/s13256-018-1637-8) contains supplementary material, which is available to authorized users.

## Background

Temporomandibular dysfunction (TMD) is a musculoskeletal disorder with many subdiagnoses, including myofascial pain, temporomandibular joint (TMJ) inflammatory conditions (traumatic, arthritides, or ankylosis), and disc derangement. TMD is often viewed as a repetitive motion disorder of the structures of mastication, with many similarities to musculoskeletal disorders of other parts of the body, and is therefore treated with similar therapeutic approaches [1]. The practitioner managing the therapy should decide which therapies are evidence based, most cost-effective, and have the greatest potential to provide long-term resolution [1]. Often a combination of splinting, physical therapy, medication therapy, and behavior modification are first-line conservative strategies [2–4]. Effective physical therapy treatment should consist of multifaceted treatment strategies, including manual techniques (that is, mobilization, stretching, and/or manipulation of the TMJ and cervical spine); exercise instruction (that is, self-stretching and mobility strategies for the TMJ and cervical spine); patient education (that is, postural instruction, relaxation techniques, and parafunctional awareness); and modalities that improve tissue health [1, 3]. In our experience, this multifaceted treatment philosophy has been successful in a majority of patients. However, occasionally there is an underlying pathology that conservative strategies cannot fully resolve. In these cases, a multidisciplinary approach with oral and/or other specialties is required.

Pathology that may require a more intensive approach is anterior disc derangement with or without reduction, secondary to posterior ligament insufficiency. These ligamentous injuries can be either acute or chronic, and most commonly result in anterior/anteromedial disc displacement due to elongation of the posterior discal ligaments. Chronic ligamentous injuries generally represent chronic instability from failure of acute ligament healing, often involving fiber stretching or tearing and joint laxity, and may have an inflammatory component [5]. Restoring the mechanics of the affected region, reducing inflammation when possible, and restoring stability are important first-line conservative measures. The combination of splinting, physical therapy, medication therapy, and behavior modification as first-line conservative strategies has been reported as effective in up to 86% of these patients with disc displacement who become pain-free and regain acceptable function with this strategy [4]. Once the environment has been optimized, symptoms are more likely to resolve; however, in stubborn cases, there may be an argument for the use of platelet-rich plasma (PRP) to synergistically assist the inflammatory cascade and regenerative processes in healing injured tissue.

PRP is a concentrate of platelets and associated growth factors obtained through withdrawal and centrifugation of a sample of a patient’s own blood [6]. Autologous platelet preparations have demonstrated potential to modify the natural healing pathway of tendons and ligaments [6]. Recently, PRP has become more widely used, especially in athletes; however, the use for TMD has not been widely studied.

## Case presentation

A 33-year-old white woman presented with a history of left-sided TMD. The dysfunction initiated when she was tackled during a football game when she was approximately 13 years of age. Symptoms originally presented as an asymptomatic click, which became painful and progressively deteriorated, eventually resulting in a sustained period where she was unable to attain full opening. She presented with an anterior disc derangement with reduction typically occurring early in the morning. It was postulated that she was bruxing at night, causing the derangement. She continued to function relatively well during the course of the day, which was somewhat atypical given the pathology present.

She attended a course of physical therapy, which provided short-term benefit, but returned 20 months later with more frequent clicking, pain, and an inability to self-manage following dental procedures. The procedures were for fillings, occurring 2 weeks apart prior to her return. She reported initial swelling in her left jaw, slurring of words due to swelling, and inability to open her mouth. The slurring resolved over the first 24 hours; however, she reported the onset of headaches into the left temporal and suboccipital region the next day. Ibuprofen was used for symptom management. She reported that her left TMJ “popped” 3 days later. She reported severe pain with the reduction; however, there was improvement in movement and pain.

This individual was now presenting with progressive disc derangement tendencies with likely further development of posterior ligament insufficiency. She also appeared to be developing degenerative changes in this joint with progressively increasing pain and movement loss. Range of motion was limited to 25 mm of opening (defined as maximal opening of incisor distance), 1 to 2 mm protrusion, 7 mm left deviation, and 7 mm right deviation with left TMJ pain reported. There was no deviation of her jaw with opening. Significant capsular stiffness was noted with all TMJ mobilizations. There was increased tone of her masseter (left > right) and lateral pterygoid (left > right). Scalenes, upper trapezius, and levator scapulae musculature (left) demonstrated increased tone with decreased length. Isometric resisted testing of all jaw muscles aggravated her left TMJ. A cervical scan demonstrated C2/3 hypomobility with flexion and extension, with normal craniovertebral (CV) ligament and vertebrobasilar artery (VBA) stress testing.

Following her second course of manual treatment, she presented with full range of motion of her TMJ, and improvement in myofascial tone. Her cervical dysfunction resolved. However, she continued to have increased frequency of clicking in her left TMJ, occasionally leading to the onset of pain. Despite improvement in range of motion and mechanics, she continued to present with a reduction click at end range of opening. The crisp and loud nature of the reduction click indicated a viable posterior ligament and reduction of the anterior displaced disc. Various options were discussed, including intermittent treatment for derangements, which had been successful in the past, or referral to an oral surgeon for consideration of disc resection and/or arthrocentesis. She was not an exceptionally good candidate for arthrocentesis as she was not fibrosed at the present time, had excellent mobility, and had a disc derangement with reduction. She was a candidate for PRP therapy, although the potential fibrosis of the posterior ligament and the viability was suspect. Due to the question of the viability of the ligament, she was educated and given 20% odds of success, but wished to proceed.

### Differential diagnosis, investigations, and treatment

Following her initial assessment, she attended a course of five treatment sessions approximately once weekly (Additional file 1: Physical Therapy Protocol). Following this course of treatment, she presented with full opening and full movement in all planes. There was no deviation of her mandible. The accessory movements of her TMJ did not indicate any derangement of the intra-articular disc. She was asymptomatic and fully functioning. The situation had resolved to its status prior to her acute crisis with an early morning click followed by a relatively asymptomatic day.

The options of management were discussed with our patient, which could have included intermittent physical therapy during crisis or PRP injection. The posterior ligament was still healthy enough to consider a distractive splint with PRP injection of the posterior ligament to induce another degree of fibrosis. However, this would require a significantly large occlusal splint that most would find quite bothersome. After considerable discussion, it was decided that she could function well enough with intermittent treatment. She was discharged with the advice to be in contact for intermittent physical therapy, as it would be likely that she would have intermittent derangement and she should seek early rather than later care.

**Fig. 1:**
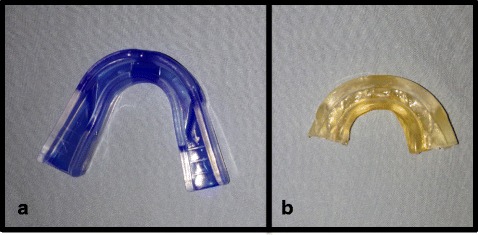
With no guiding protocol relative to splinting post-platelet-rich plasma injection, we used a large plastic athletic splint Brain-Pad LoPro Double Laminated Strap/Strapless Combo in one Adult Mouthguard **(a)** to keep the disc in a reduced position at 15 mm of opening for 24 hours a day. The immobilization period was for 3 weeks, followed by a gradual weaning period with reduced posterior support **(b)** for an additional 5 weeks

For the second course of treatment, she received three additional physical therapy treatment sessions, consisting of biomechanical optimization of her cervical spine and TMJ, leaving her again with full range of motion of her TMJ, and improvement in myofascial tone. Her cervical dysfunction resolved. However, she continued to have increased frequency of clicking in her left TMJ, occasionally leading to the onset of pain. She opted for PRP injection, and given that there was no guiding protocol relative to splinting, we planned to use a large plastic athletic splint Brain-Pad LoPro Double Laminated Strap/Strapless Combo in one Adult Mouthguard (Fig. 1a) to keep the disc in a reduced position at 15 mm of opening for 24 hours a day. The exception was for dental care, which would be done with the mouth in a fully open position, avoiding closure. The immobilization period was for 3 weeks, followed by a gradual weaning period with reduced posterior support (Fig. 1b) for an additional 5 weeks.

### Injection procedure

The PRP injection procedure was performed by a chronic pain specialist, with the assistance of a physical therapist and nurse. Sixty ml of whole blood was withdrawn from the antebrachial area. The Magellan centrifuge (Arteriocyte Medical Systems, Hopkinton, MA, USA) was used to obtain a total of 4 ml of PRP. Acetaminophen 1000 mg and 50 mg of tramadol were provided as pre-procedure analgesics.

The physical therapist present assisted in examination and localizing the site of injection based on anatomic landmarks and tenderness. The physical therapist manually reduced the anterior disc derangement with a combination of distraction and anterior glide until the mandibular condyle was under the anteriorly displaced disc. This was maintained through the injection process by compression under the angle of the mandible, squeezing the disc condyle relationship to the eminence. The skin and superficial tissues were anesthetized with 1% lidocaine. Ultrasound guidance was used to introduce the PRP into the center of the temporal fossa and distribute the PRP at angles centrally, posteriorly, and anteriorly into the posterior ligament. A total of 3 ml of PRP was injected into the involved structures with a 27 gauge, 1.5 inch needle. Without closing the mouth, the splint was put in place. Our patient was advised to keep the appliance in 24 hours a day for 3 weeks, with the exception of dental care which was to be done with the mouth in a fully open position, avoiding closure. She was instructed to use a liquid diet, as well as concepts of appropriate nutrition and oral hygiene. To maximize benefits, she was given advice about PRP injection rehabilitation. Post-injection pain over the next few days was treated with acetaminophen or tramadol, with avoidance of anti-inflammatories for 4 weeks.

### Outcome and follow-up

**Fig. 2:**
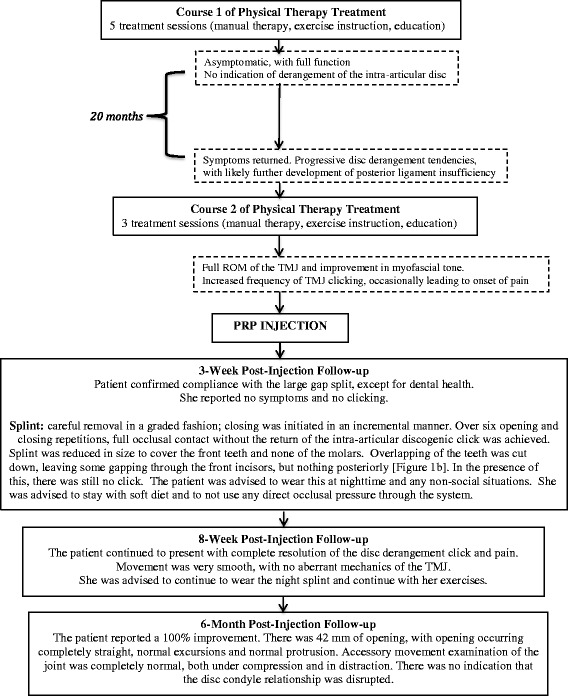
Timeline of initial physical therapy management, platelet-rich plasma injection, and post-procedure physical therapy follow-up and associated outcomes. PRP: platelet-rich plasma, ROM: range of motion, TMJ: temporomandibular joint

She was followed up by a physical therapist at 3 weeks, 8 weeks, and 6 months post-injection. Follow-up procedures and outcomes are outlined in Fig. 2.

## Discussion

Conservative management of physical therapy, consisting of multifaceted treatment strategies, including manual techniques, exercise instruction, and patient education was initially successful in this case. However, it was not sufficient to prevent recurrent symptoms. Because of the insufficiency of the posterior ligament, symptoms would return. Although the effects of the physical therapy treatment were not long lasting, this was a crucial component to be performed pre-injection, in order to obtain maximal longitudinal length of the capsule to allow maximal disc motion without compression.

Occlusal splints alone can be used as temporary and conservative treatment; however, our patient would have to maintain the 15 mm open gap splint for a year or more to stabilize and provide pain relief with some patients reporting no improvement even with up to 9 years of follow-up [7]. Prolonged use of repositioning appliances for internal disc derangement and/or osteoarthritis can cause undesirable and irreversible changes in dental occlusion, skeletal structure, and muscle dynamics [8].

## Conclusions

In this case, the interdisciplinary approach was prudent to fully resolve this TMD efficiently and effectively. The result of this case was exceptionally successful, with our patient reporting 100% improvement 6 months post-injection. Prior to this case, we predicted a 20% success rate, based on her inability to maintain the effects of conservative management over the long term. We are confident that this protocol will be more successful than expected, even in more chronic cases, as long as there is viability of the posterior ligament based on clinical examination. The outcome of this case provides evidence to warrant further research.

## Additional file

Additional file 1: Components of physical therapy protocol pre-injection. (DOCX 18 kb)

## Abbreviations

CV: Craniovertebral
PRP: Platelet-rich plasma
TMD: Temporomandibular dysfunction
TMJ: Temporomandibular joint
VBA: Vertebrobasilar artery
