# Supplementary material for: Anterior disc derangement with reduction of the temporomandibular joint: a case report
Source: J Med Case Rep. 2018 May 27;12:148. doi: 10.1186/s13256-018-1637-8 (PMC5971088; doi:10.1186/s13256-018-1637-8)
Supplement: Supplementary file 1 — Components of physical therapy protocol pre-injection. (DOCX 18 kb) [file 13256_2018_1637_MOESM1_ESM.docx]

Additional file 1 Components of physical therapy protocol pre-injection

| **MANUAL THERAPY** |
| --- |
| **JOINT MOBILIZATION** |
| Distraction mobilization to lengthen the capsule |
| Anterior/posterior glide |
| Lateral/medial glide |
| Functional opening |
|  |
| **MYOFASICAL MOBILIZATION** |
| Masseter |
| Temporalis |
| Lateral pterygoid |
| Medial pterygoid |

* Optimizing mechanics of the joint should be attained prior to injection, with the above mobilization techniques. Capsule lengthening with a distraction mobilization is crucial.

| **EXERCISE INSTRUCTION** | **EDUCATION** |
| --- | --- |
| **PHASE 1: Flexibility of the TMJ and cervical spine** | - Habit modification - Diet modification - Proper positioning of the TMJ - Application of moist heat or ice to side of face - Relaxation techniques - Postural education |
| Opening stretch |  |
| Lateral stretch |  |
| Protrusion stretch |  |
| Retrusion stretch |  |
| Upper trapezius stretch |  |
| Scalene stretch |  |
| Myofascial stripping of masseter, temporalis, lateral and medial pterygoid |  |
| **PHASE 2: Strength, stability, and proprioception** |  |
| Isometric strengthening in neutral |  |
| Isometric strengthening at mid-range |  |
| Isometric strengthening at end-range |  |
| Isotonic strengthening |  |
| Opening proprioception |  |
| Controlled opening |  |
| End of range opening |  |
| Postural correction |  |
